# Spatially resolved lipid compartmentalization in human pancreatic islets revealed by high-resolution ultra-low-flow-rate desorption electrospray ionization mass spectrometry imaging

**DOI:** 10.1007/s00216-026-06557-4

**Published:** 2026-05-22

**Authors:** Wan-Jo Lee, Thao Duong, Hua Zhang, Deep Kapadia, Peter Chlebeck, Kelly H. Lu, Connie S. Chamberlain, Jon S. Odorico, Lingjun Li

**Affiliations:** 1 Department of Chemistry, University of Wisconsin-Madison, Madison, WI 53706 USA; 2 School of Pharmacy, University of Wisconsin-Madison, Madison, WI 53705 USA; 3 Department of Surgery, University of Wisconsin–Madison, 600 Highland Ave, Madison, WI 53792 USA

**Keywords:** Mass spectrometry imaging, u-DESI-MSI, Human pancreatic islets, Lipidomic analysis, MALDI-MSI, Spatial distribution

## Abstract

Human pancreatic islets are highly heterogeneous; thus, understanding their biological organization is crucial for elucidating metabolic function and diabetes pathogenesis. High-resolution mass spectrometry imaging of intact human pancreas is challenging due to the small size and dispersed distribution of individual islets within dense exocrine tissue. Here, we establish an ultra-low-flow-rate DESI mass spectrometry imaging (u-DESI-MSI) platform that enables lipidomic analysis of individual islets in human pancreatic tissue. Optimization of raster step size and scan rate parameters for u-DESI resulted in lipid ion images with enhanced spatial fidelity. The analysis demonstrates a highly reproducible central–peripheral spatial lipid distribution within pancreatic tissue. Diacyl phosphatidylcholines (diacyl-PCs), ether-linked phosphatidylcholines (ether-linked PCs) and sphingomyelins (SMs) are predominantly localized to the central endocrine region and co-register with intact insulin distributions, as further validated by matrix-assisted laser desorption/ionization mass spectrometry imaging (MALDI-MSI). In contrast, specific lysophosphatidylcholines (LPCs) delineate the endocrine–exocrine interface. These results indicate the existence of position-dependent lipid organization within human islets. This platform provides a robust spatial-mapping foundation for future studies investigating structural and metabolic alterations in human islets under disease conditions.

**Graphical abstract:**

**Figure:**
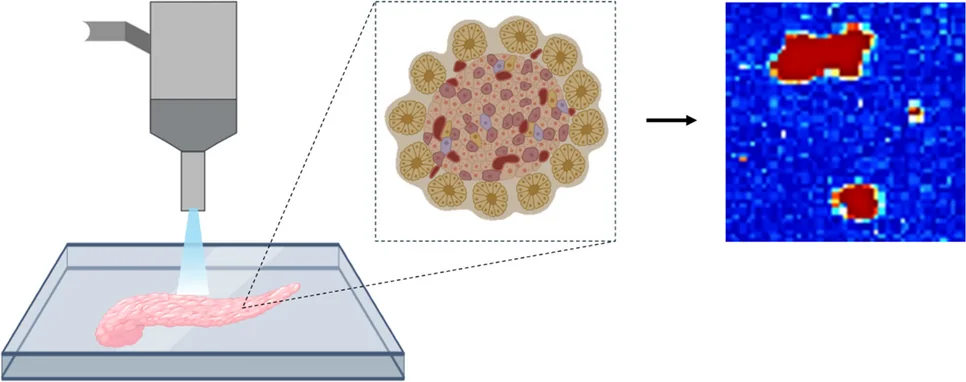

**Supplementary Information:**

The online version contains supplementary material available at https://doi.org/10.1007/s00216-026-06557-4.

## Introduction

The pancreas is a multifunctional organ that integrates exocrine digestion with endocrine glucose regulation, the latter being governed by the islets of Langerhans—highly specialized micro-organs composed of insulin-secreting β-cells, glucagon-secreting α-cells, somatostatin-secreting δ-cells, and additional endocrine and stromal cell types [1]. Although islet biology has been extensively studied at the cellular level, accurately resolving molecular heterogeneity within intact human pancreatic tissue remains technically challenging due to the small size, dispersed distribution, and complex lipid-rich environment of individual islets [2, 3]. Human islets are embedded within a densely packed exocrine matrix, where endocrine and exocrine compartments are tightly interwoven, making precise spatial delineation difficult [4–6]. Furthermore, the high abundance of structural lipids and overlapping biochemical signals obscure the distinct chemical boundaries required for compartment-specific analysis in situ [7]. Conventional bulk homogenization–based lipidomics and proteomics average molecular signals across the entire pancreas, masking critical heterogeneity between endocrine and exocrine compartments as well as among individual islets [8–12]. Complementary mass spectrometry–based studies have demonstrated molecular heterogeneity within pancreatic islets across different cellular and molecular scales [13, 14]. However, these methods typically use dissociated cells and lack precise spatial context in intact tissue. Mass spectrometry imaging (MSI) overcomes this limitation by enabling label-free, in situ molecular mapping directly from tissue sections [15]. For example, matrix-assisted laser desorption/ionization mass spectrometry imaging (MALDI-MSI) has been applied to mouse and human pancreas to resolve islet-specific phospholipids and gangliosides with intra-islet spatial resolution, highlighting localized oxidative stress and β-cell vulnerability [16]. However, MALDI-MSI typically requires matrix application and vacuum compatibility, and matrix-related background in the low *m/z* range can complicate sample preparation and limit detection of certain lipid and metabolite classes [17, 18]. Ambient ionization techniques, particularly desorption electrospray ionization (DESI) and nanospray DESI (nano-DESI), address several of these constraints by enabling direct analysis of unmodified tissue under atmospheric conditions with minimal sample pretreatment [19, 20]. DESI-based MSI provides rapid, label-free detection of lipids and metabolites using soft ionization, preserving labile species and permitting subsequent histological staining. It is especially advantageous for profiling low-mass metabolites that are often obscured by matrix background in MALDI [21]. Recent technological advances in nano-DESI and ultra-low-flow-rate DESI (u-DESI) platforms have enabled near single-cell and even subcellular spatial resolution, substantially reducing analyte delocalization, improving sample confinement, and enhancing spatial fidelity beyond that achievable with conventional DESI-MSI approaches [22–25]. Recent nano-DESI-MSI studies have demonstrated high-spatial resolution mapping of mouse pancreas, revealing individual islets [23]. Despite these advances, a systematic application of DESI-MSI to structurally intact human pancreatic tissue at single-islet resolution has not yet been achieved due to persistent technical constraints [16, 23, 26, 27].

In this work, we establish high-resolution u-DESI-MSI as a platform capable of resolving lipid distributions at the single-islet level in human pancreas. This approach enables direct characterization of islet spatial architecture and lipid microenvironments, providing new spatially informed insights into lipid organization within intact human pancreatic islets.

## Materials and methods

### Chemicals and reagents

Paraformaldehyde (PFA, 32% wt/vol aqueous solution, methanol-free) was purchased from Electron Microscopy Sciences. Poly-L-lysine solution (0.1% w/v) was purchased from Ted Pella. α-Cyano-4-hydroxycinnamic acid (CHCA) and tert-butyl methyl ether (MTBE) were purchased from Sigma-Aldrich. Phosphate-buffered saline (PBS, 1 ×), ammonium acetate, carboxymethylcellulose sodium salt (CMC), Triton X-100, formic acid and bovine serum albumin (BSA; Lampire Biologicals) were obtained from Fisher Scientific. Rabbit monoclonal anti-glucagon antibody was purchased from Abcam (Cat. No. ab92517). Optima LC/MS-grade acetonitrile, water, methanol, ethanol (200 proof, molecular biology grade), 2-propanol (IPA, HPLC grade), ProLong™ Gold Antifade Mountant with DAPI (Cat. No. P36931), guinea pig anti-insulin polyclonal antibody (Cat. No. PA129383), goat anti-guinea pig highly cross-adsorbed Alexa Fluor 488 secondary antibody (Cat. No. A11073) and goat anti-rabbit highly cross-adsorbed Alexa Fluor 568 secondary antibody (Cat. No. A11036) were obtained from Thermo Fisher Scientific. Peptide Calibration Standard II (700–3200 Da, Cat. No. NC1162644) was obtained from Bruker Daltonics via Fisher Scientific.

### Tissue preparation for mass spectrometry imaging

Human pancreatic tissues were obtained from the University of Wisconsin Organ and Tissue Donation Program (UW Health, Madison, WI) and Versiti Organ and Tissue Donation Services (Milwaukee, WI) from donors without a documented history of diabetes or pancreatitis. Research authorization was provided by next of kin in accordance with institutional guidelines. Tissues were received within 24 h of recovery, trimmed to remove extra-pancreatic tissue and stored at − 80 °C. The tissues were thawed and fixed in paraformaldehyde (PFA) and embedded in 2% (w/v) carboxymethyl cellulose (CMC). Tissue blocks were cryosectioned at 20 μm using a cryostat (Leica CM3050 S, Leica Microsystems, Germany). Sections were mounted onto indium tin oxide (ITO)-coated glass microscope slides (25 mm × 75 mm × 1.1 mm, Delta Technologies, Loveland, CO, USA) that were pre-coated with poly-L-lysine and stored at − 20 °C before analysis. For u-DESI-MSI analysis, sections were removed from storage, thawed at room temperature, and dried in a desiccator for 30 min. For MALDI-MSI analysis, sections were thawed and dried, then washed using a series of solvent steps adapted from published protocols [28]. Briefly, sections were sequentially immersed in 70% ethanol for 30 s, 100% ethanol for 30 s, rinsed twice in distilled water, then washed again in 70% ethanol for 30 s and 100% ethanol for 30 s. After washing, sections were dried at room temperature for about 15 min before matrix application. The α-Cyano-4-hydroxycinnamic acid (CHCA) matrix was prepared at 10 mgmL^−1^ in acetonitrile/water (70:30, v/v). Matrix was applied using an M5 Sprayer (HTX Technologies, Chapel Hill, NC, USA) at a flow rate of 100 μL min^−1^, with a track spacing of 3 mm and 8 passes.

### High-resolution u-DESI-MSI acquisition

u-DESI-MSI experiments were conducted using a Waters cyclic ion mobility quadrupole time-of-flight mass spectrometer equipped with a DESI-XS ion source (Waters, Milford, MA). Data were acquired in positive ion mode over an *m/z* range of 50–1200. Spatial resolution was controlled by adjusting the raster step size between 10 and 50 μm depending on the experimental objective. A nanoAcquity UPLC system (Waters) was employed to supply the spray solvent (96% methanol, 4% water, 0.1% formic acid) at a flow rate of 0.3 μL/min. The solvent was introduced through a 30 μm inner-diameter metal emitter coupled to a 400 μm gas nozzle assembly supplied with nitrogen gas at 0.8 bar. The solvent was delivered through a PolyGLYCOPLEX A capillary column (3 μm, 0.15 × 150 mm; PolyLC Inc.) prior to nebulization. The emitter was positioned at an incidence angle of 70° relative to the sample surface, with a 0.5 mm sprayer-to-surface distance and approximately 5 mm separation between the sprayer tip and MS inlet. The capillary voltage was optimized between 0.6 and 1.0 kV, and the cone voltage was set to 60 V. The heated inlet capillary was maintained at 120 °C and positioned 0.5 mm above the tissue surface. For structural verification, selected lipid ions were subjected to on-tissue MS/MS analysis under transfer collision energy of 35 V, with resolution settings of 15 (low mass) and 20 (high mass). The instrumental configurations and operational parameters for high-resolution u-DESI-MSI were adopted and modified from previously established protocols [24]. Lipid assignments obtained from u-DESI-MSI were further corroborated by offline LC-nanoESI-MS/MS analysis of lipid extracts.

### MALDI-MSI acquisition

MALDI-TOF MSI was performed on a rapifleX MALDI-TOF/TOF instrument (Bruker Daltonics, Bremen, Germany) in linear positive-ion mode over an *m/z* range of 2000–7000. The laser spot diameter was 30 μm, with 90% laser power, 300 laser shots per pixel, and a 10 kHz repetition rate. External calibration was performed using a Bruker Peptide Calibration Standard II spotted on the same slide before tissue imaging. Instrument control and data acquisition used FlexControl and FlexImaging software (Bruker Daltonics).

### H&E staining and immunofluorescence staining

Hematoxylin and eosin (H&E) staining was performed on tissue sections after u-DESI-MSI and MALDI-MSI analysis to provide histological context. For MALDI-MSI samples, tissue sections were washed twice with 70% ethanol for 30 s to remove the matrix prior to staining. H&E staining was subsequently performed by the Translational Research Initiatives in Pathology (TRIP) laboratory (Department of Pathology and Laboratory Medicine, University of Wisconsin-Madison, Madison, WI) using standard protocols. Immunofluorescence (IF) staining was performed directly on the same tissue sections following u-DESI-MSI analysis. For IF staining, tissue sections were circled with a hydrophobic barrier pen. Sections were washed three times with 1 × PBS, permeabilized with 0.05% Triton X-100 in PBS for 15 min, and blocked with 10% BSA in PBS for 35 min at room temperature. Primary antibody incubation was performed sequentially. Sections were incubated overnight at 4 °C with a glucagon primary antibody diluted in 1% BSA/PBS, followed by incubation with the goat anti-rabbit highly cross-adsorbed Alexa Fluor 568 secondary antibody diluted in 1% BSA/PBS for 40 min at room temperature in the dark. After another blocking step, sections were incubated with an insulin primary antibody for 1 h at room temperature, followed by incubation with the goat anti-guinea pig highly cross-adsorbed Alexa Fluor 488 secondary antibody for 40 min in the dark. Sections were mounted using ProLong Gold antifade mounting medium containing DAPI and stored in the dark before imaging. H&E and immunofluorescence images were acquired using an Olympus microscope with a DP75 digital camera and appropriate halogen and fluorescence light sources. Image acquisition and processing were performed using cellSens software.

### Data processing and statistical analysis

Raw MSI data were imported into SCiLS Lab software (Bruker Daltonics, Bremen, Germany) for processing and visualization. All ion images were normalized to total ion current (TIC). For MALDI-MSI, insulin ion signals defined islet regions of interest (ROIs), and islet morphology was confirmed by H&E staining. These insulin-positive areas served as a spatial reference for defining ROIs in the subsequent u-DESI-MSI analysis. For u-DESI-MSI data, lipid ion peaks were manually selected based on spectral features and signal intensity. Tentative lipid identifications were assigned by matching *m/z* values with LC–nanoESI–MS/MS results. u-DESI-MS/MS data were processed using Waters HDI 1.6 software with a 0.02 Da *m/z* window and a 3000 MS resolution to generate averaged *m/z* spectra for structural validation. To characterize the spatial heterogeneity within pancreatic islets, ion images of specific lipid markers were generated and overlaid using SCiLS Ion Image Mapper (Bruker Daltonics) software. For quantitative analysis, ROIs representing the islets were defined within u-DESI-MSI datasets guided by the reference images, and pixel-level ion intensities were extracted. Statistical comparisons of relative ion intensities at selected *m/z* values were performed using SCiLS Lab.

### Lipid extract analysis via LC-nanoESI-MS/MS

Total lipids were extracted from pancreas tissues using the methyl tert-butyl ether (MTBE) method [29]. Approximately 10 mg of each sample was homogenized in 40 mL of 0.1% ammonium acetate with a handheld probe sonicator, employing a 24-s cycle (8 s on, 15 s off) at 50% amplitude. Subsequently, 1 mL of MTBE was added, and the mixture was incubated for 15 min at room temperature on a shaker. Phase separation was induced by adding 150 µL of MS-grade water, followed by a 10-min incubation at room temperature. Samples were centrifuged at 1,000 g for 10 min, and the upper organic layer was collected. The remaining lower phase was re-extracted with 0.4 mL of a solvent matching the upper phase composition (MTBE/methanol/water, 10:3:2.5, v/v/v), incubated for 15 min, and centrifuged at 1,000 g for 10 min. The upper organic layers were pooled and dried using a vacuum centrifuge. High-performance liquid chromatography-nanoelectrospray ionization tandem mass spectrometry (HPLC-nanoESI-MS/MS) analysis was performed on a Q-Exactive Orbitrap mass spectrometer (Thermo Scientific, San Jose, CA) coupled with a Waters NanoAquity UPLC system. Chromatographic separation and nanoESI emission were achieved using a custom microcapillary column (75 μm ID) packed with 12 cm of bridged ethylene hybrid C18 particles (1.7 μm, Waters). Mobile phase A consisted of acetonitrile (ACN)/water (40/60, v/v), and mobile phase B consisted of isopropanol (IPA)/ACN (90/10, v/v), both containing 10 mM ammonium acetate. Samples were reconstituted in 100 µL of a 50:50 mixture of mobile phase A and mobile phase B. The sample solution was centrifuged at 10,000 g for 10 min, and the supernatant was analyzed by liquid chromatography-nanoelectrospray ionization tandem mass spectrometry (LC-nanoESI-MS/MS). Lipids were separated over a 133-min gradient elution at a flow rate of 200 nL/min: 0–22 min, 5% B; 22–23 min, 5–30% B; 23–28 min, 30–35% B; 28–33 min, 35–51% B; 51–58 min, 51–61% B; 58–68 min, 61–70% B; 68–78 min, 70–99% B; 78–93 min, 99% B; 93–94 min, 99–60% B; 94–97 min, 60% B; 98–103 min, 99% B; 103–108 min, 99%–5% B; 108–133 min, 5% B. Mass spectra were acquired over an *m/z* range of 200–1200 (positive ion mode) with an ionization voltage of ± 2.0 kV and a capillary temperature of 275 °C. Survey scans were conducted at a resolving power of 70,000 (at *m/z* 200), an automatic gain control (AGC) target of 1 × 10^6^, and a maximum injection time of 250 ms. The top 15 precursors were selected for MS/MS data acquisition. MS/MS analysis used an isolation window of 2.0 Da over the *m/z* range 200–1200, an AGC target of 1 × 10^5^, and a maximum injection time of 150 ms. High-energy collisional dissociation (HCD) was performed on charge states 2–7 with normalized collision energy (NCE) set to 25%. All other mass spectrometry parameters were set to default values. Pancreas lipidomics data were analyzed using MSDIAL (version 4.9).

## Results and discussion

### Optimization of sample preparation and u-DESI-MSI acquisition parameters

To gain a deeper understanding of the distribution of islets in human pancreatic tissues using high-resolution u-DESI-MSI, we systematically optimized key acquisition parameters. Prior to refining the imaging conditions, we briefly evaluated the impact of tissue preservation methods using MALDI-MSI targeting the hormone insulin. As shown in Figure S1, fresh frozen tissue embedded in 2% CMC yielded superior spatial resolution compared to formalin-fixed paraffin-embedded (FFPE) tissue. This performance difference is primarily attributed to formaldehyde-induced crosslinking in FFPE tissues, which leads to the formation of methylene bridges between the nucleophilic sites on biomolecules such as peptides, proteins, and DNA [30, 31]. Such fixation process can impede the efficient desorption and ionization of biomolecules during MSI, particularly larger molecular species such as proteins and the hormone insulin. Therefore, 2% CMC-embedded frozen tissue was selected for all subsequent u-DESI-MSI experiments to ensure optimal preservation of native biomolecular profiles.

**Fig. 1:**
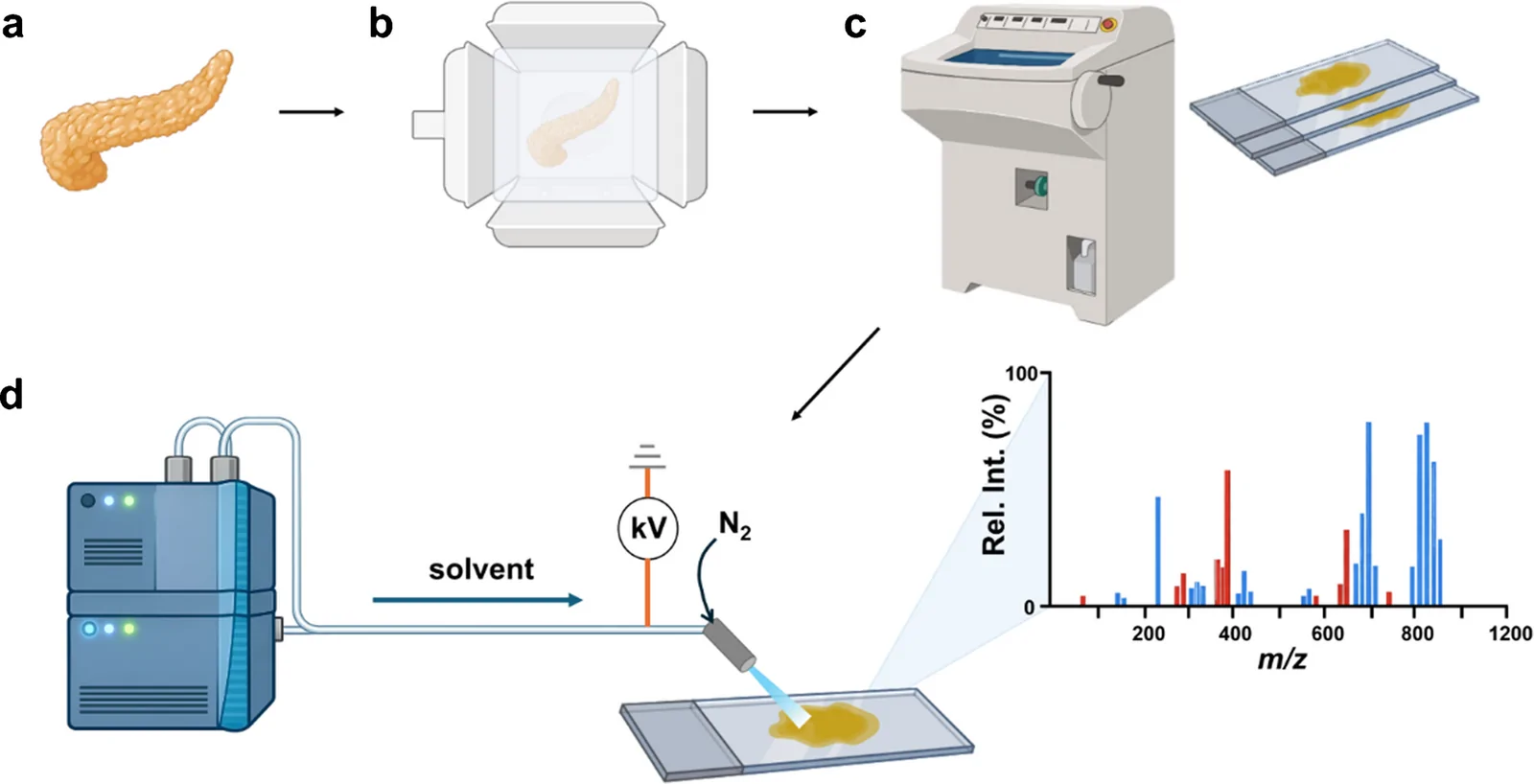
Workflow of the developed method for u-DESI-MSI analysis of pancreatic tissue:** a** Human pancreatic tissue; **b** Tissue embedded in 2% CMC; **c** Tissue cryosectioned at 20 μm using a cryostat and mounted onto poly-L-lysine pre-coated ITO glass slides; **d** u-DESI-MSI analysis performed after storage at − 20 °C

**Fig. 2:**
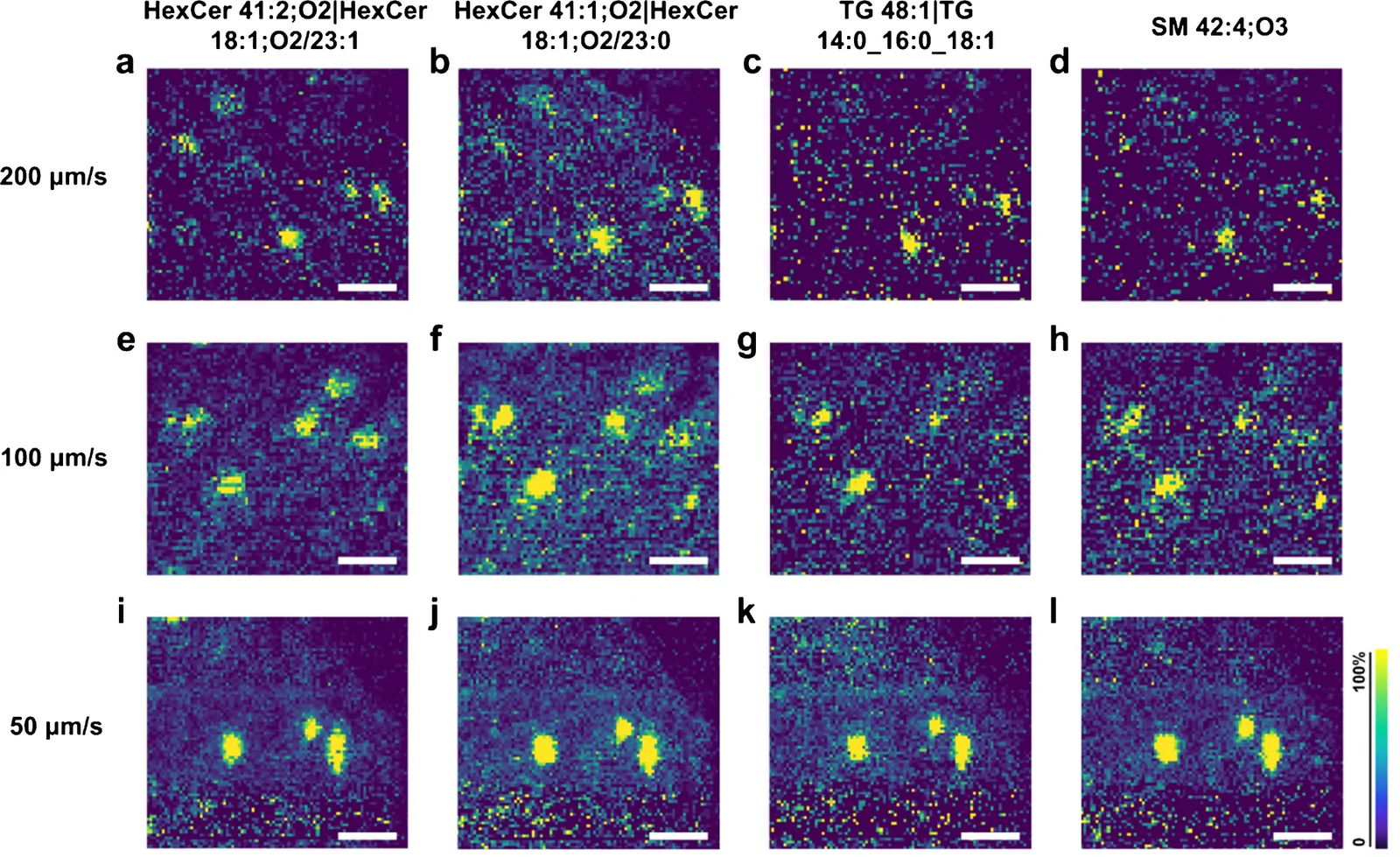
u-DESI-MSI of human pancreatic tissue with scan rates decreased from 200 μm/s to 50 μm/s.** a**–**d** Representative ion images of HexCer 41:2;O2|HexCer 18:1;O2/23:1 (*m/z* 796.063), HexCer 41:1;O2|HexCer 18:1;O2/23:0 (*m/z* 798.082), TG 48:1|TG 14:0_16:0_18:1 (*m/z* 822.031), and SM 42:4;O3 (*m/z* 825.023) acquired at a scan rate of 200 μm/s; **e**–**h** Corresponding lipid species acquired at a scan rate of 100 μm/s; **i**–**l** Corresponding lipid species acquired at a scan rate of 50 μm/s. Scale bar is 500 μm; all u-DESI-MSI images were acquired with a mass error tolerance of 10 ppm

As illustrated in the overall experimental workflow (Fig. 1), human pancreatic tissues were cryosectioned at 20 μm thickness and directly analyzed by u-DESI-MSI. To achieve stable and efficient lipid desorption, the DESI source was coupled to an M-Class LC system, which delivered solvent at a flow rate of 0.3 μL/min. Concurrently, nitrogen gas was supplied at 0.8 bar to facilitate the nebulization process. Under the optimized settings, we first examined the influence of different scan rates. For image quality assessment, we selected representative high-abundance lipids (such as HexCer, TG, and SM species) that exhibited well-defined spatial patterns. Figure 2 shows that a fast scan rate of 200 μm/s resulted in reduced spatial resolution and diminished signal intensity. Lowering the scan rate significantly improved the spatial resolution and markedly improved image clarity. At the optimal scan rate of 50 μm/s, spatial resolution was substantially enhanced, enabling clear visualization of regions enriched in high-abundance lipids.

**Fig. 3:**
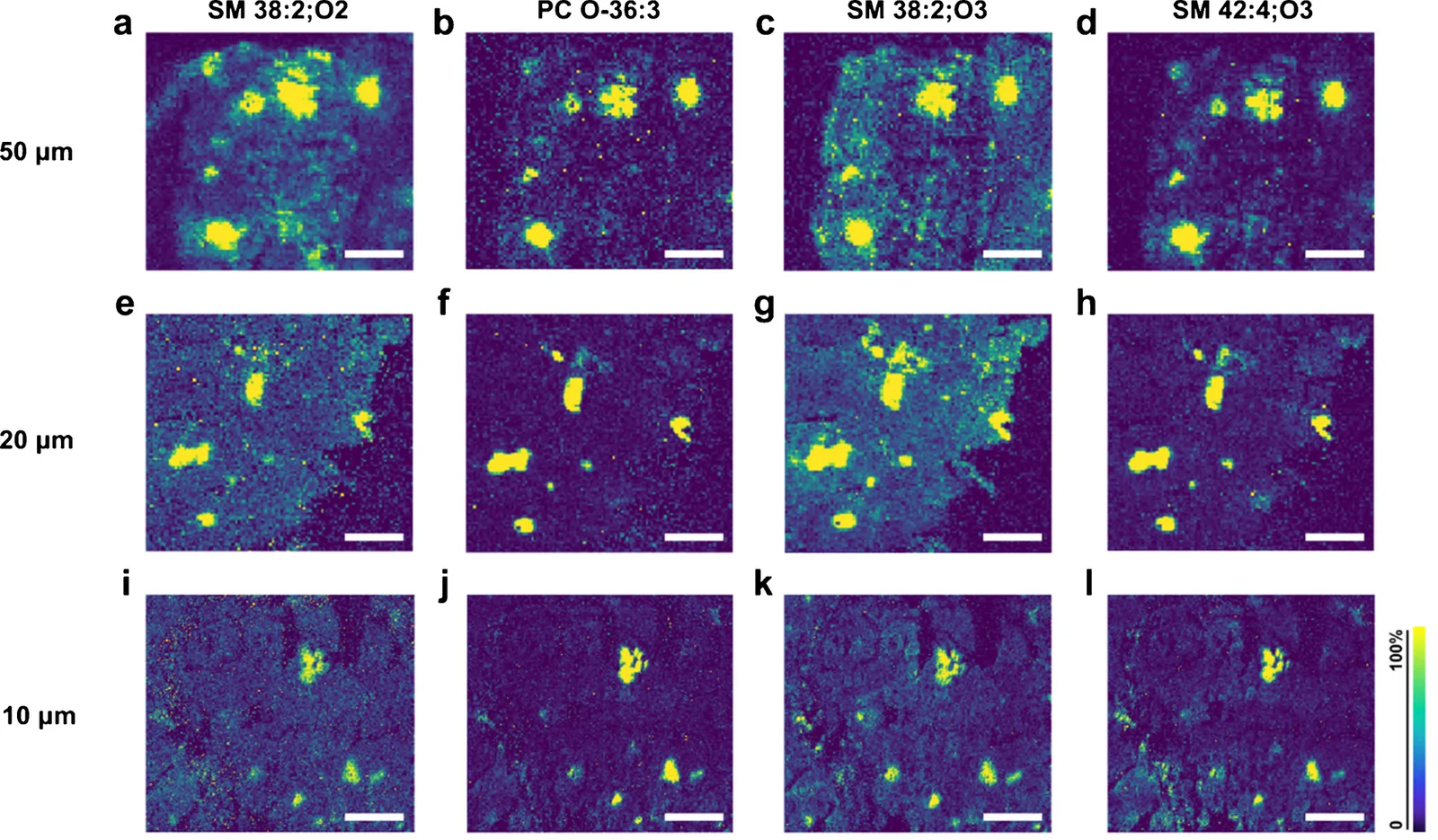
u-DESI-MSI of human pancreatic tissue with step size decreased from 50 μm to 10 μm.** a**–**d** Representative ion images of SM 38:2;O2 (*m/z* 757.214), PC O-36:3 (*m/z* 770.119), SM 38:2;O3 (*m/z* 773.129), and SM 42:4;O3 (*m/z* 825.023) acquired at a step size of 50 μm. **e**–**h** Corresponding lipid species acquired at a step size of 20 μm. **i**–**l** Corresponding lipid species acquired at a step size of 10 μm. Scale bar: 500 μm; all u-DESI-MSI images were acquired with a mass error tolerance of 10 ppm

Furthermore, to determine whether these high-abundance lipid regions could resolve well-defined microstructures, we evaluated different raster step sizes (Fig. 3). Using representative SMs and ether-linked PCs species as markers, we observed that the structural boundaries of high-abundance regions became substantially sharper as the step size decreased. Because a 10 µm step size greatly increased acquisition time without providing a proportionally meaningful improvement in biologically relevant spatial resolution compared with 20 µm, we selected 20 µm as the optimal step size to balance spatial resolution and throughput in our subsequent tissue mapping experiments.

### Mapping the spatial lipidomic architecture and microstructures

**Fig. 4:**
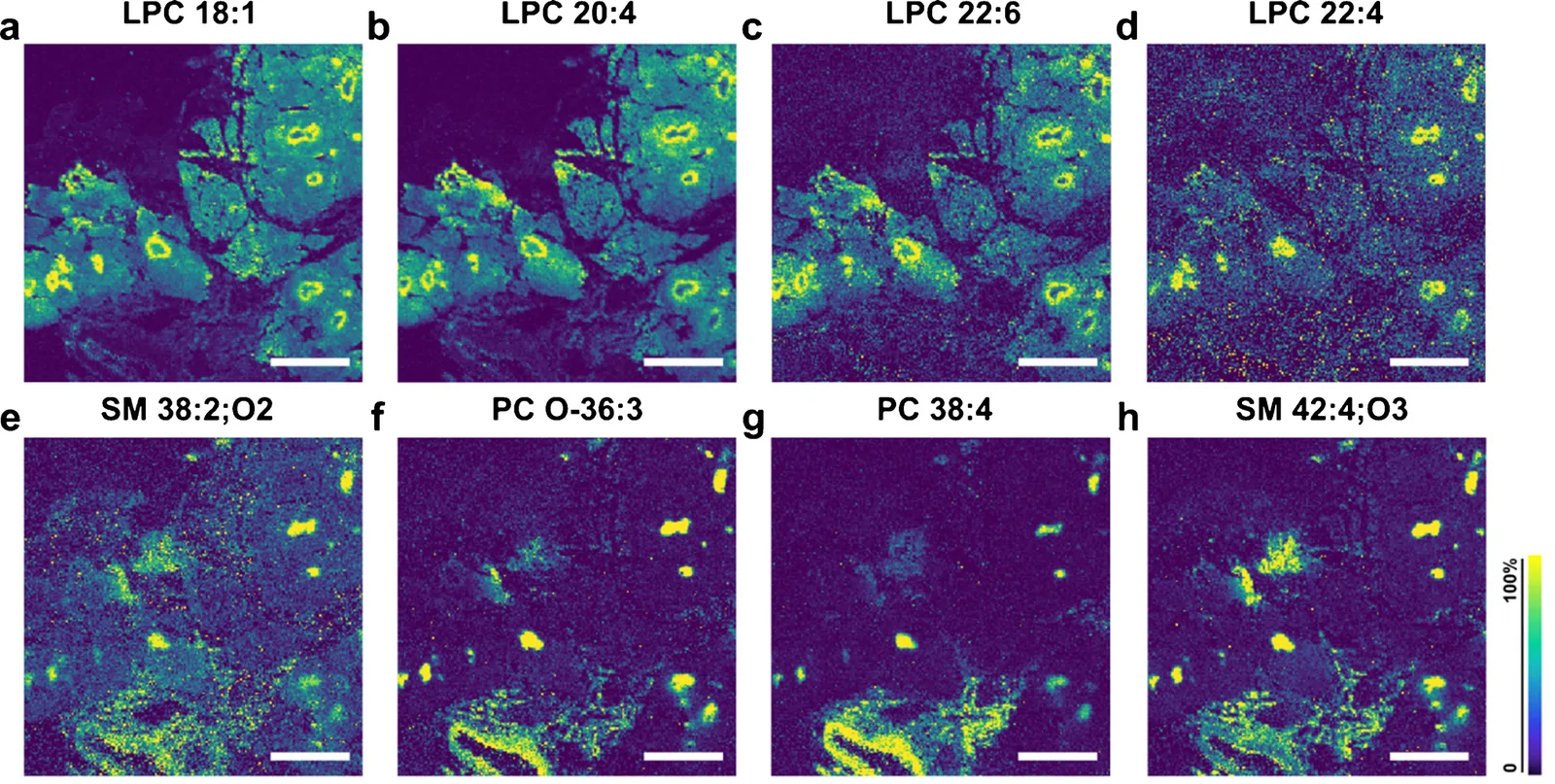
u-DESI-MSI of human pancreatic tissue acquired with a raster step size of 20 μm. **a**–**h** Representative ion images of **a** *m/z* 522.332 (LPC 18:1), **b** *m/z* 544.325 (LPC 20:4), **c** *m/z* 568.336 (LPC 22:6), **d** *m/z* 572.368 (LPC 22:4), **e** *m/z* 757.592 (SM 38:2;O2), **f** *m/z* 770.556 (PC O-36:3), **g** *m/z* 810.657 (PC 38:4), and **h** *m/z* 825.601 (SM 42:4;O3). These representative ion images were acquired at a scan rate of 40 μm/s using an M-Class LC system with a pump flow rate of 0.3 μL/min. Scale bar is 1 mm; all u-DESI-MSI images were obtained with a mass error tolerance of 10 ppm

Using the optimized scan rate and step size described above, we acquired high-resolution u-DESI-MSI data across a large area of human pancreatic tissue. We utilized a step size of 20 μm and a scan rate of 40 μm/s to balance spatial resolution with signal-to-noise ratio. To map the lipidome, we screened for high-abundance lipid features in the *m/z* 500–900 range. As shown in Fig. 4, the results revealed significant spatial heterogeneity among different lipid classes. Based on the lipid assignments obtained from our LC- nanoESI-MS/MS data (Table S1), we identified distinct spatial patterns in the high-resolution u-DESI-MSI ion images. The structural identities of representative lipid markers were further confirmed by on-tissue u-DESI-MS/MS (Figure S2). Several lower-mass LPCs (*m/z* 500–600), such as LPC 18:1 (*m/z* 522.332), LPC 20:4 (*m/z* 544.325), and LPC 22:6 (*m/z* 568.336), formed ring-like structures that clearly delineated the boundaries of discrete microregions within the surrounding exocrine acini. In contrast, higher-mass lipids (*m/z* 700–900), mainly diacyl-PCs, ether-linked PCs and SMs, exhibited a different distribution pattern. For example, PC O-36:3 (*m/z* 770.556), PC 38:4 (*m/z* 810.657), and SM 42:4;O3 (*m/z* 825.601) were predominantly localized to the central region of these rings. These circular microregions, about 50–250 μm in diameter, are consistent with the reported size range of human pancreatic islets [4, 32].

**Fig. 5:**
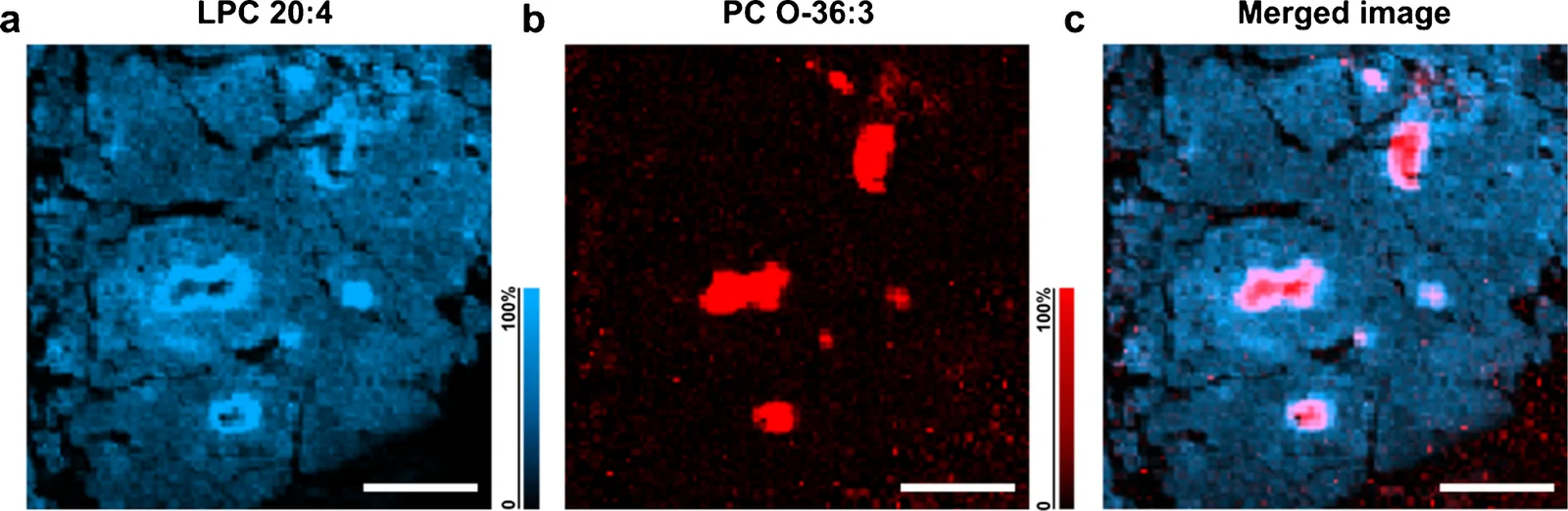
Zoomed-in u-DESI-MSI images visualizing selected regions of interest (ROI) in pseudocolor. **a** LPC 20:4 (*m/z* 544.325) displayed in blue. **b** PC O-36:3 (*m/z* 770.556) displayed in red. **c** Two-color merged image demonstrating spatial complementarity. Scale bar, 500 μm

To further investigate the microarchitecture of these regions, we performed ion image co-registration and overlay analysis using SCiLS Lab software (Fig. 5). By merging ion images of a peripherally enriched lipid (LPC 20:4, shown in blue) with those of a centrally enriched lipid (PC O-36:3, shown in red), we observed a clear central–peripheral spatial organization. LPC 20:4 primarily surrounded the PC O-36:3-rich center, with a modest degree of overlap in the peripheral region, visualized as a pink signal. This suggests a gradual spatial transition between the central and peripheral lipid microregions rather than a sharply defined boundary. This spatial segregation was highly reproducible across numerous microregions within the tissue, supporting the interpretation that it reflects a consistent biological feature rather than a technical artifact. The central regions were enriched in diacyl-PCs, ether-linked PCs and SMs, whereas LPCs were more abundant at the periphery, demonstrating a clear lipid subclass-specific spatial distribution. This organized molecular architecture may represent previously unrecognized compositional heterogeneity within these human islet-like structures. However, visual interpretation of lipid patterns alone cannot definitively establish the anatomical identity of these microregions. Therefore, before performing targeted region-of-interest (ROI) quantification, we sought to confirm their endocrine identity by mapping intact insulin using MALDI-TOF MSI.

### Validation of islet microstructures via MALDI-MSI and histology

To verify that the observed islet-like microregions were actual islet structures in pancreatic tissue, we performed MALDI-MSI on the same tissue section that had been analyzed by u-DESI-MSI (Figure S3a, c). Using the same section allowed direct comparison of lipid and insulin/peptide distributions at each pixel, avoiding alignment errors commonly encountered with serial sections. Before performing MALDI-MSI, the section was washed with ethanol and water to remove lipid, small metabolite, and salt residues remaining after u-DESI-MSI [27]. This step reduced ion suppression and improved the ionization of larger peptide species [33, 34]. Using this protocol, we mapped the spatial distribution of the hormone insulin (*m/z* 5821.46), a definitive marker of pancreatic β-cells [16]. Following MALDI-MSI acquisition, hematoxylin and eosin (H&E) staining provided morphological confirmation that these circular microregions correspond to islets of Langerhans embedded within the surrounding exocrine tissue (Figure S3b).

**Fig. 6:**
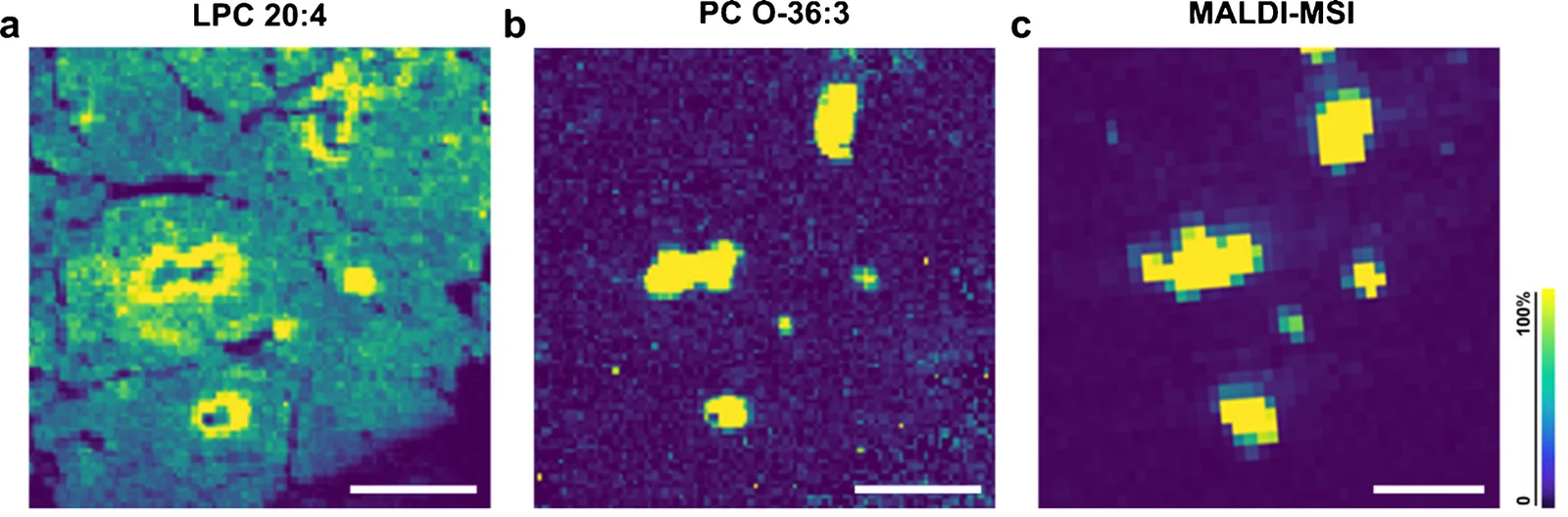
Validation of spatial distributions using u-DESI-MSI and MALDI-MSI on the same human pancreatic tissue section. Representative lipid u-DESI-MSI images of **a** LPC 20:4 (*m/z* 544.325) and **b** PC O-36:3 (*m/z* 770.556). **c** Representative MALDI-MSI image of the hormone insulin (*m/z* 5821.46) acquired from the same tissue. Scale bar: 500 μm; all MSI images were obtained with a mass error tolerance of 10 ppm

Figure 6 shows that the lipid species mapped by u-DESI-MSI and the hormone insulin mapped by MALDI-MSI are found within the same region. The MALDI-MSI signal for insulin (Fig. 6c) matches the central distribution of the ether-linked lipid PC O-36:3 (*m/z* 770.556) observed by u-DESI-MSI (Fig. 6b). Because insulin is found only in pancreatic islets, this central area represents the endocrine compartment [35, 36]. Surrounding this central region is a continuous peripheral lipid zone rich in specific LPC species, such as LPC 20:4 (Fig. 6a). The LPC 20:4 (*m/z* 544.325) signal localizes specifically to the endocrine-exocrine interface, delineating the central endocrine region from the surrounding exocrine tissue. This spatial pattern indicates that the tissue is highly organized, with certain lipid subclasses highlighting the interface between the exocrine and endocrine compartments. Taken together, these results confirm that the observed lipid microregions represent the unique spatial structure of human pancreatic tissue.

### Quantitative validation and cellular context of the endocrine–exocrine interface

**Fig. 7:**
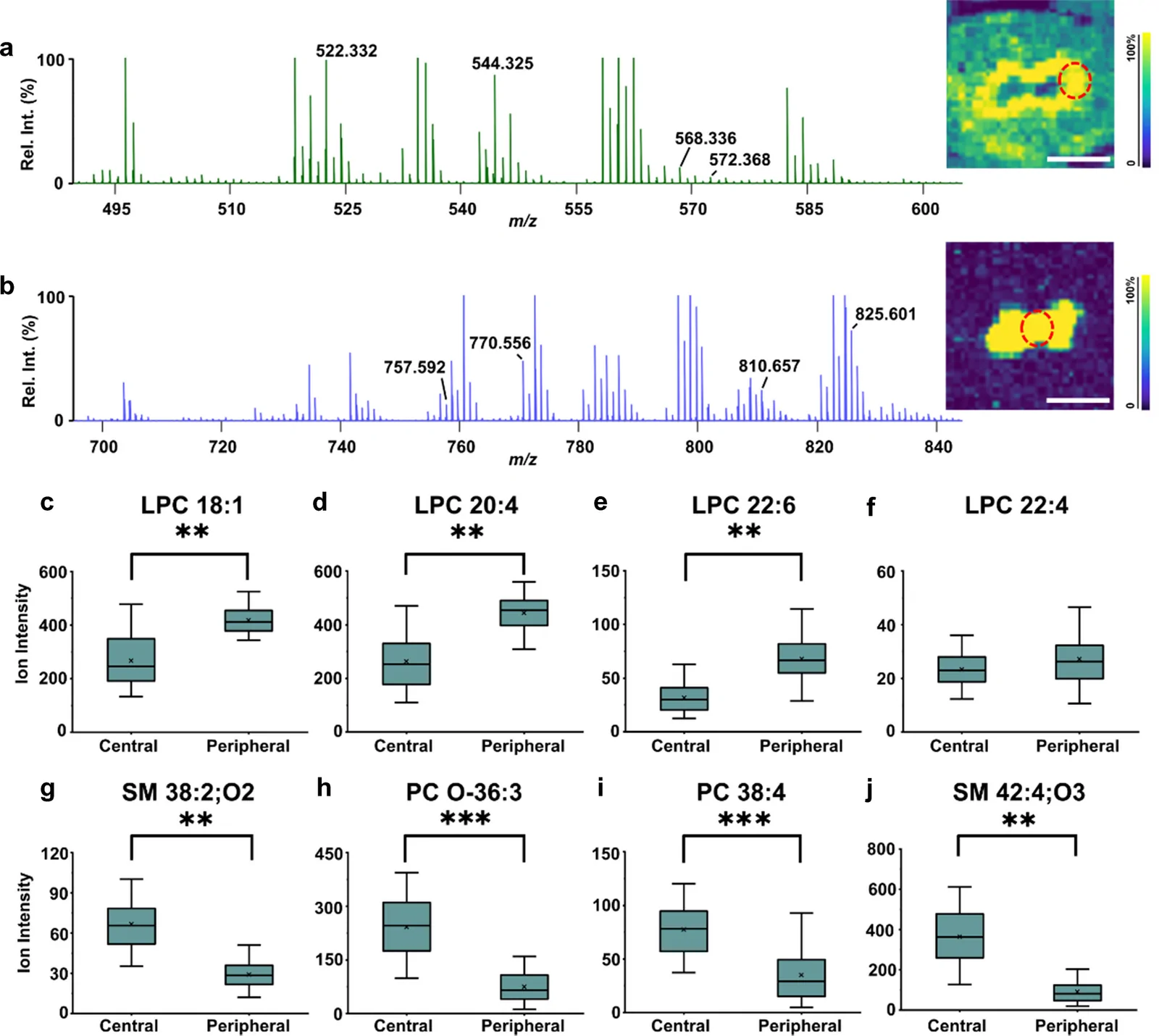
u-DESI-MSI–derived mass spectra and ion intensity comparisons of representative lipid species extracted from specific regions of interest (ROIs) within human pancreatic tissue. **a** Mass spectrum extracted from the peripheral ROI of the islet. The adjacent representative ion image (*m/z* 544.325) provides a visual reference for the corresponding spatial location. **b** Mass spectrum extracted from the central ROI of the islet. The adjacent representative ion image (*m/z* 770.556) provides a visual reference for the corresponding spatial location. **c**–**j** Box plots of ion intensities for representative lipid species comparing the central and peripheral ROIs of the islet: **c** LPC 18:1 (*m/z* 522.332), **d** LPC 20:4 (*m/z* 544.325), **e** LPC 22:6 (*m/z* 568.336), **f** LPC 22:4 (*m/z* 572.368), **g** SM 38:2;O2 (*m/z* 757.592), **h** PC O-36:3 (*m/z* 770.556), **i** PC 38:4 (*m/z* 810.657), **j** SM 42:4;O3 (*m/z* 825.601). Mass spectra represent summed signals from u-DESI-MSI pixels within the selected ROIs, and box plots represent the distribution of pixel intensities within each ROI. Statistical significance was assessed using a paired t-test across individual islets (*n* = 6). Scale bars (ion images), 250 μm. *p < 0.05, **p < 0.01, ***p < 0.001

After confirming the identity of islet structures, we set out to identify their compartment-specific lipid profiles. Using the co-registered MSI data, we defined two regions of interest (ROI): the central endocrine region of the islet and the peripheral region at the endocrine–exocrine interface. When we extracted average mass spectra from these areas (Fig. 7), we observed clear differences in their lipid compositions. The peripheral region contained lower-mass lipid species (Fig. 7a, *m/z* 500–600), whereas the central region exhibited higher-mass lipid signals (Fig. 7b, *m/z* 700–900). These results are consistent with our observation of lipid segregation between the central and peripheral regions. To further validate this difference, we selected specific islet structures and statistically compared lipid levels across these well-defined ROIs throughout the tissue (Fig. 7c–j). For example, quantitative analysis revealed that diacyl-PCs, ether-linked PCs and SMs (such as PC O-36:3, PC 38:4 and SM 42:4;O3) were significantly enriched in the central endocrine region (paired *t*-test, n = 6). In contrast, LPCs (like LPC 18:1, LPC 20:4 and LPC 22:6) exhibited higher signal intensities at the endocrine–exocrine interface. These quantitative results statistically confirm the differential abundance of these representative lipid subclasses between the central endocrine region and the peripheral region.

To determine whether these lipid profiles were related to the distribution of specific endocrine cell types, we performed immunofluorescence staining for insulin (β-cells) and glucagon (α-cells) on nearby tissue sections (Figure S4). Unlike the well-defined mantle–core arrangement observed in rodent models [2], the human islets we examined displayed a mixed distribution of α- and β-cells throughout the endocrine region [4, 32]. The sharp peripheral lipid pattern, represented by LPC 20:4, did not correspond to a distinct outer layer of α-cells. Because the lipid pattern does not align with cell-type distribution, these results suggest that lipid organization is not determined solely by endocrine cell identity. Although the spatial resolution of immunofluorescence analysis may be affected by prior MALDI-MSI acquisition [37], the overall mixed cellular pattern observed supports the conclusion that lipid compartmentalization does not directly correspond to a simple cell-type organization. Future studies incorporating alternative approaches, such as tissue expansion–assisted imaging of isolated islets [38–42], may further refine the cellular context underlying this spatial lipid organization.

## Conclusion

In summary, we developed a high-resolution u-DESI-MSI platform to map lipid organization at the single-islet level in structurally intact human pancreatic tissue. By integrating u-DESI-MSI with complementary validation methods – including MALDI-TOF MSI, LC–nanoESI–MS/MS, H&E staining, and immunofluorescence staining, we demonstrated that endocrine islets exhibit a consistent, position-dependent lipid organization characterized by distinct central and peripheral subclass distributions. Notably, this lipid pattern does not precisely mirror the mosaic arrangement of α- and β-cells, suggesting that spatial lipid organization reflects the complex microenvironment within human islets. This platform establishes a foundation for future investigations into how alterations in lipid organization may influence islet function and contribute to disease-associated alterations. Future work could apply this approach to isolated single islets to elucidate the relationship between spatial lipid patterns, cellular identity, and diabetes-associated alterations. In addition, integrating complementary techniques such as ion mobility separation may further enhance understanding of lipid subclass complexity and isomeric diversity within the islet microenvironment.

## Supplementary Information

Below is the link to the electronic supplementary material.

Supplementary file1 (DOCX 2.84 MB)

## Data Availability

All data supporting the findings of this study are available within the article and its Supplementary Information. Raw mass spectrometry imaging datasets and additional file formats are available from the corresponding author upon reasonable request.

## Declarations

Human pancreatic tissues were obtained from the University of Wisconsin Organ and Tissue Donation Program (UW Health, Madison, WI) and Versiti Organ and Tissue Donation Services (Milwaukee, WI) from donors without a documented history of diabetes or pancreatitis. Research authorization was provided by next of kin in accordance with Institutional Review Board guidelines.
