# Supplementary material for: Spatially resolved lipid compartmentalization in human pancreatic islets revealed by high-resolution ultra-low-flow-rate desorption electrospray ionization mass spectrometry imaging
Source: Anal Bioanal Chem. 2026 May 22;418(20):6605–17. doi: 10.1007/s00216-026-06557-4 (PMC13574116; doi:10.1007/s00216-026-06557-4)
Supplement: Supplementary file 1 [file 216_2026_6557_MOESM1_ESM.docx]

***Supporting Information***

**Spatially Resolved Lipid Compartmentalization in Human Pancreatic Islets Revealed by High-Resolution Ultra-Low-Flow-Rate Desorption Electrospray Ionization Mass Spectrometry Imaging**

Wan-Jo Lee^1#^, Thao Duong^1#^, Hua Zhang^2^*, Deep Kapadia^3^, Peter Chlebeck^3^, Kelly H. Lu^1^, Connie S Chamberlain^3^, Jon S Odorico^3^, and Lingjun Li^1, 2^*

^1^Department of Chemistry, University of Wisconsin-Madison, Madison, Wisconsin 53705, United States

^2^School of Pharmacy, University of Wisconsin-Madison, Madison, Wisconsin 53705, United States

^3^Department of Surgery, University of Wisconsin–Madison, 600 Highland Ave, Madison, WI 53792, USA

\# These authors contributed equally: Wan-Jo Lee and Thao Duong.

**Corresponding authors**

Correspondence to

Hua Zhang: [hzhang7.lab@gmail.com](mailto:hzhang7.lab@gmail.com)

Lingjun Li: [lingjun.li@wisc.edu](mailto:lingjun.li@wisc.edu)

**Table of Content**

**Figure S1.** Comparison of MALDI-MSI between FFPE and fresh frozen human pancreatic tissues

**Figure S2.** Representative u-DESI-MS/MS spectra of selected lipid species detected in human pancreatic tissue

**Figure S3.** MALDI-MSI and histological staining of human pancreatic tissue

**Figure S4.** Immunofluorescence staining of human pancreatic tissue performed after u-DESI-MSI analysis

**Table S1.** List of identified lipid species from human pancreatic samples analyzed in positive ion mode

**Figure S1. Comparison of MALDI-MSI between FFPE and fresh frozen human pancreatic tissues.** Representative ion images of the hormone insulin from **a** an FFPE human pancreatic tissue (*m/z* 5821.39) and **b** a fresh-frozen human pancreatic tissue embedded in 2% CMC (*m/z* 5821.46). Scale bar, 1 mm; all MALDI-MSI images were obtained with a mass error tolerance of 10 ppm.

**Figure S2. Representative u-DESI-MS/MS spectra of selected lipid species detected in human pancreatic tissue, showing characteristic fragment ions that support structural assignment.**

**Figure S2*. (Continued) Representative u-DESI-MS/MS spectra of selected lipid species detected in human pancreatic tissue, showing characteristic fragment ions that support structural assignment.**

**Figure S3. MALDI-MSI and histological staining of human pancreatic tissue. a** Representative MALDI-MSI image of the hormone insulin (*m/z* 5821.46) **b** Optical image of H&E staining performed on the same tissue section after MALDI-MSI analysis, with selected islet regions indicated by black circles. **c** Representative MALDI mass spectrum acquired from the tissue. Scale bars, 500 μm.

**Figure S4. Immunofluorescence (IF) staining of human pancreatic tissue performed after u-DESI-MSI analysis.** Representative u-DESI-MS ion images of **a** LPC 20:4 (*m/z* 544.325) and **b** PC O-36:3 (*m/z* 770.556). **c**-**e** Representative IF staining images acquired from the same tissue section showing **c** glucagon (red), **d** insulin (green), and **e** the overlaid image. Scale bars, 50 μm.

**Table S1. List of identified lipid species from human pancreatic samples analyzed in positive ion mode**

| **Name** | **Molecular Formula** | **Measured mass (m/z)** | **Exact mass (m/z)** | **Type** | **Mass error (ppm)** |
| --- | --- | --- | --- | --- | --- |
| LPC 18:1 | C26H52NO7P | 522.3553 | 522.3554 | [M+H]^+^ | -0.23 |
| LPC O-20:2 | C28H56NO6P | 534.3919 | 534.3918 | [M+H]^+^ | 0.07 |
| LPC 20:4 | C28H50NO7P | 544.3394 | 544.3398 | [M+H]^+^ | -0.42 |
| LPC 22:6 | C30H50NO7P | 568.3404 | 568.3398 | [M+H]^+^ | 0.61 |
| LPC 22:4 | C30H54NO7P | 572.3707 | 572.3711 | [M+H]^+^ | -0.36 |
| SM 36:0;O2 | C41H85N2O6P | 733.6216 | 733.6218 | [M+H]^+^ | -0.19 |
| PC 32:0 | C40H80NO8P | 734.5691 | 734.5694 | [M+H]^+^ | -0.31 |
| PC 34:4 | C42H76NO8P | 754.5462 | 754.5381 | [M+H]^+^ | 8.11 |
| SM 38:2;O2 | C43H85N2O6P | 757.6214 | 757.6218 | [M+H]^+^ | -0.43 |
| PC 34:2 | C42H80NO8P | 758.5696 | 758.5694 | [M+H]^+^ | 0.18 |
| SM 38:1;O2 | C43H87N2O6P | 759.6378 | 759.6375 | [M+H]^+^ | 0.25 |
| PC O-36:3 | C44H84NO7P | 770.6066 | 770.6058 | [M+H]^+^ | 0.86 |
| PC O-36:2 | C44H86NO7P | 772.6204 | 772.6215 | [M+H]^+^ | -1.10 |
| SM 38:2;O3 | C43H85N2O7P | 773.6176 | 773.6167 | [M+H]^+^ | 0.91 |
| PC 36:5 | C44H78NO8P | 780.5535 | 780.5538 | [M+H]^+^ | -0.24 |
| PC 36:4 | C44H80NO8P | 782.5640 | 782.5694 | [M+H]^+^ | -5.44 |
| PC 36:3 | C44H82NO8P | 784.5849 | 784.5851 | [M+H]^+^ | -0.18 |
| PC 36:2 | C44H84NO8P | 786.6001 | 786.6007 | [M+H]^+^ | -0.61 |
| HexCer 41:2;O2\|HexCer 18:1;O2/23:1 | C47H89NO8 | 796.6688 | 796.6661 | [M+H]^+^ | 2.68 |
| HexCer 41:1;O2\|HexCer 18:1;O2/23:0 | C47H91NO8 | 798.6842 | 798.6817 | [M+H]^+^ | 2.50 |
| PC 38:7 | C46H78NO8P | 804.5465 | 804.5538 | [M+H]^+^ | -7.32 |
| PC 38:5 | C46H82NO8P | 808.5856 | 808.5851 | [M+H]^+^ | 0.55 |
| SM 42:4;O2 | C47H89N2O6P | 809.6537 | 809.6531 | [M+H]^+^ | 0.61 |
| PC 38:4 | C46H84NO8P | 810.6013 | 810.6007 | [M+H]^+^ | 0.61 |
| PC O-40:6 | C48H86NO7P | 820.6210 | 820.6215 | [M+H]^+^ | -0.55 |
| TG 48:1\|TG 14:0_16:0_18:1 | C51H96O6 | 822.7542 | 822.7545 | [M+NH_4_]^+^ | -0.37 |
| PC O-40:4 | C48H90NO7P | 824.6537 | 824.6528 | [M+H]^+^ | 0.92 |
| SM 42:4;O3 | C47H89N2O7P | 825.6476 | 825.6480 | [M+H]^+^ | -0.37 |
| PC 40:8 | C48H80NO8P | 830.5691 | 830.5694 | [M+H]^+^ | -0.31 |
| TG 50:3\|TG 16:0_16:1_18:2 | C53H96O6 | 846.7565 | 846.7545 | [M+NH_4_]^+^ | 1.95 |
| TG 50:2\|TG 16:0_16:1_18:1 | C53H98O6 | 848.7705 | 848.7702 | [M+NH_4_]^+^ | 0.25 |

*****Error = ((experimental mass - theoretical adduct mass)/ theoretical adduct mass)*1000000
